# Experimental warming and precipitation interactively modulate the mortality rate and timing of spring emergence of a gallmaking Tephritid fly

**DOI:** 10.1038/srep32284

**Published:** 2016-08-31

**Authors:** Xinqiang Xi, Dongbo Li, Youhong Peng, Nico Eisenhauer, Shucun Sun

**Affiliations:** 1 Department of Ecology, School of Life Sciences, Nanjing University, 163 Xianlindadao Avenue, Nanjing 210023, China; 2 ECORES Lab, Chengdu Institute of Biology, Chinese Academy of Sciences, Chengdu 610041, China; 3 German Centre for Integrative Biodiversity Research (iDiv) Halle-Jena-Leipzig, Deutscher Platz 5e, 04103 Leipzig, Germany; 4 Institute for Biology, Leipzig University, Johannisallee 21, 04103 Leipzig, Germany

## Abstract

Global climate change is mostly characterized by temperature increase and fluctuating precipitation events, which may affect the spring phenology and mortality rate of insects. However, the interaction effect of temperature and precipitation on species performance has rarely been examined. Here we studied the response of the gall-making Tephritid fly *Urophora stylata* (Diptera: Tephritidae) to artificial warming, changes in precipitation, and the presence of galls. Our results revealed a significant interaction effect of warming, precipitation, and galls on the life-history traits of the focal species. Specifically, when the galls were intact, warming had no effect on the phenology and increased the mortality of the fly under decreased precipitation, but it significantly advanced the timing of adult emergence and had no effect on the mortality under increased precipitation. When galls were removed, warming significantly advanced the timing of emergence and increased fly mortality, but precipitation showed no effect on the phenology and mortality. In addition, gall removal significantly increased adult fresh mass for both females and males. Our results indicate that the effect of elevated temperature on the performance of species may depend on other environmental conditions, such as variations in precipitation, and species traits like the formation of galls.

Climate change may significantly alter the performance of various species^1^,^2^. One well-known response of organisms to climate change is the shift in species phenology, as indicated by the advance of vegetation greening and animal emergence in spring^3^. This response is particularly important to many herbivorous insect species because spring phenology determines the duration of their growth and development in the following growing season as well as their synchrony with the phenology of host plants (e.g. ref. 4). Another significant effect of climate change is on species mortality rate, which is exemplified by frequent pest outbreaks in several recent reports^5^,^6^. In particular, in temperate regions, where the major environmental fluctuations during the transition period from winter to spring often result in high insect mortality rate^6^, a slight change in spring climate can be crucial to population dynamics of insects. Studies have used historical records^7^ or laboratory data on thermal tolerance^8^ to infer the effect of climate change on insect survival and phenology. However, field experiments are still largely lacking (but see ref. 9), which may limit the accuracy of predictions of insect survival and phenology in nature-like conditions^6^.

Global warming may shift animal spring phenology^10^ because temperature is critical to animal metabolic rate and activity particularly for ectothermic insects whose physiological processes are largely temperature-dependent^11^. Spring warming therefore likely advances the emergence of hibernating insects in temperate regions^12^. Consistently, numerous studies have recorded the advance of spring emergence in various insect species^13^,^14^,^15^. Furthermore, increasing temperatures may also alter insect mortality^16^,^17^ by physiologically alleviating cold stress and shortening the period of insects living below the lethal temperature threshold^18^,^19^. This has been demonstrated in many insect species including moth (*Thaumetopoea pityocampa*)^20^, aphid (*Myzus persicae*)^21^, and butterfly (*Atalopedes campestris*)^22^. However, warming does not always lead to an advance in phenology and increase survival of insects. For example, neutral and negative effects of warming on spring phenology (delayed) and survival have also frequently been reported, varying with locations and species^23^,^24^,^25^,^26^. These contrasting results suggest that warming effects might be mediated by other abiotic factors and/or species traits^26^.

One of the potential co-determining abiotic factors of species survival and phenology is the change in precipitation. In fact, in addition to shifts in precipitation patterns, extreme precipitation events are likely to become more frequent in many parts of the world^27^,^28^. Newly emerged insect bodies are often particularly sensitive to air humidity and soil moisture^26^. For example, at high temperatures the newly emerged insects may die of excessive water loss if water availability is too low^29^, whereas excessive precipitation can induce high mortality due to microbial pathogens^30^,^31^. Possibly because of such a close relationship between precipitation and mortality, spring emergence has been shown to not only depend on temperature but also to be associated with the amount of precipitation in many insect species^24^. However, few studies have simultaneously manipulated both temperature and precipitation to explore their interaction effects on insect survival and phenology.

Species traits are likely to mediate responses of insect species to global warming. Insects may undergo unfavorable winters in different life stages (e.g. as larvae, pupae, or adults) with different strategies against environmental fluctuations (with none, with ootheca, silk bags or plant galls covered eggs or larvae^26^). For instance, naked larvae should be more sensitive to temperature change than those embedded in galls, which are often produced to prevent parasitoid enemies in many insect species^32^. Galls do not only block light to make insects insensitive to photoperiod but also buffer external temperature fluctuations^32^,^33^. Typically, insects can ‘sense’ physical environmental change and emerge from galls unless the galls are moistened by precipitation^32^. Thus, we hypothesize that gall-making species are less likely to be sensitive to changes in temperature than in precipitation.

The Tibetan plateau has experienced pronounced warming during the past decades and is predicted to have a higher than average temperature increase in the near future^34^,^35^, and extreme precipitation events occur more frequently than before^35^. Climate change has induced a significant change in plant phenology^36^ and primary production^37^ on the plateau, and these changes can be ascribed to temperature increase or altered precipitation. Yet, effects of increasing temperature and altered precipitation on insect phenology and survival have rarely been tested in this high altitudinal region. In this study, we investigated the effects of experimental warming and manipulated precipitation on the mortality and spring emergence of the gall-making Tephritid fly *Urophora stylata* (Diptera: Tephritidae). The primary question was whether the warming effect on the life history traits of the studied insect species depends on precipitation. In addition, we also experimentally removed galls to examine the gall effect on species mortality and phenology. The second question was whether the climate effect on species performance is modulated/mediated by the presence of galls.

## Results

### Mortality rate

**Figure 1:**
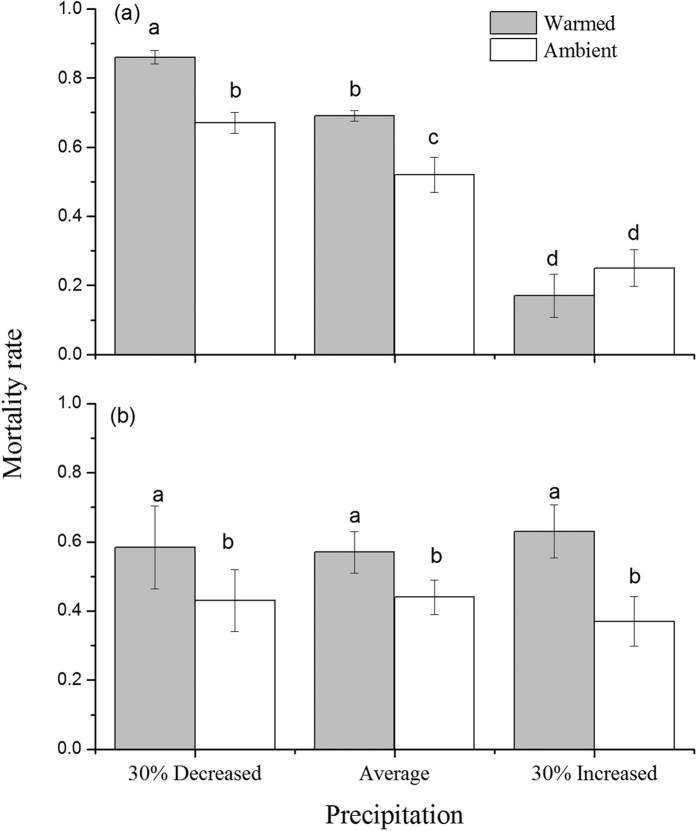
Mortality rate of larval maggots with intact (**a**) and with galls removed (**b**) as affected by warming (ambient, warmed (ambient +1.9 °C)) and precipitation (30% increased precipitation, average (average of past 53 years), and 30% decreased precipitation). Different letters above the bars denote significant differences among treatments at *P* = 0.05.

Warming and precipitation as well as precipitation and galls interactively affected the mortality rate of larvae (Table 1). The presence of galls significantly decreased the mortality rate of the maggots (Table 1, Fig. 1). When galls were intact, the mortality rate decreased with increasing precipitation (Table 1, Fig. 1a). Moreover, warming significantly increased the mortality rate of flies in the decreased and average precipitation treatments but did not significantly alter mortality at increased precipitation (significant warming × precipitation interaction; Table 1, Fig. 1a). When the galls were removed, warming consistently increased mortality rates in all of the precipitation treatments, while the precipitation effect was non-significant (Fig. 1b).

### Timing of adult emergence and adult fresh mass

**Figure 2:**
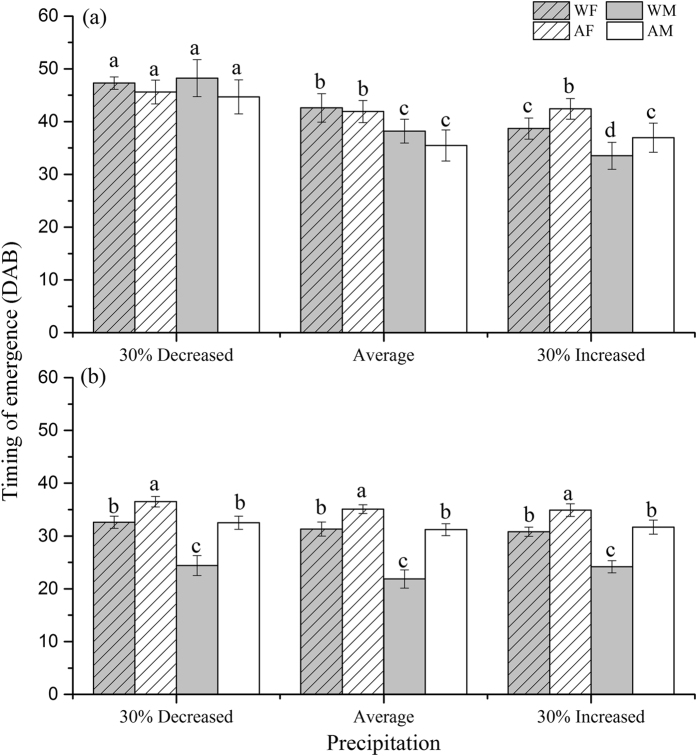
Timing of emergence (as reflected by the days after the beginning of the experiment; DAB) of adults developed from larval maggots with intact (**a**) and with galls removed (**b**) as affected by warming (ambient, warmed (ambient +1.9 °C)), precipitation (30% increased precipitation, average (average of past 53 years), and 30% decreased precipitation) and sex (AM: ambient male; AF: ambient female; WM: warmed male; WF: warmed female). Different letters above the bars denote significant differences among treatments at *P* = 0.05.

Warming, precipitation, and galls interactively affected the timing of adult emergence (significant warming × precipitation × gall interaction; Table 2). When galls were intact, warming delayed the adult emergence for males but not females under average precipitation condition, but it advanced the adult emergence for both sexes under increased precipitation (Fig. 2a).

When galls were removed, warming significantly advanced the adult emergence for both sexes (Fig. 2b), but males (5.3 days in advance) emerged significantly earlier than females (3.1 days advance; Table 2). The precipitation effect, however, was not significant (Fig. 2b). Moreover, the effect of galls on the timing of adult emergence was significant (Table 2), as indicated by the earlier emergence of adults (by 13.2 days on average) in the gall-removed than gall-intact treatment (Fig. 2).

**Figure 3:**
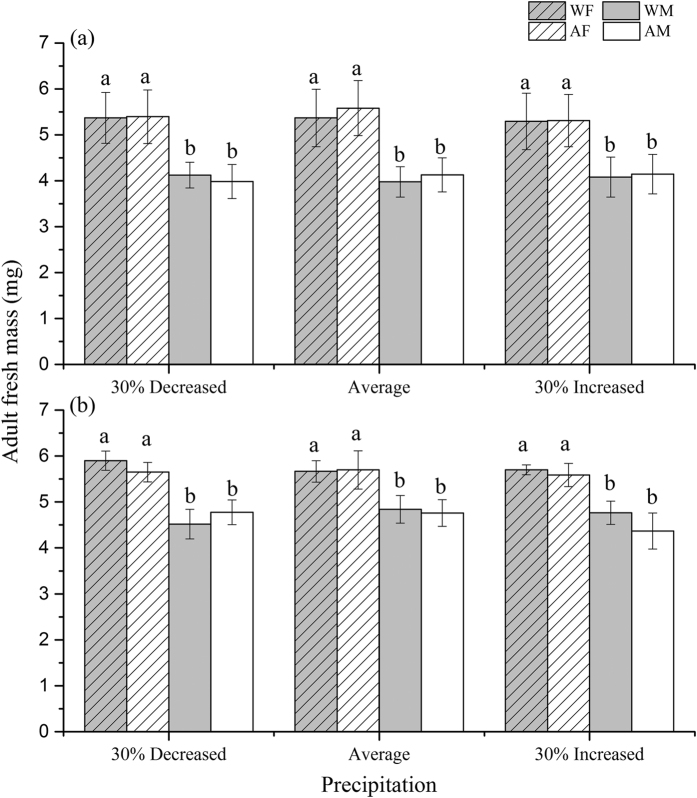
Fresh mass of adults emerged from larval maggots with intact (**a**) and with galls removed (**b**) as affected by warming (ambient, warmed (ambient +1.9 °C)), precipitation (30% increased precipitation, average (average of past 53 years), and 30% decreased precipitation), the presence of galls (intact, removed) and sex (AM: ambient male; AF: ambient female; WM: warmed male; WF: warmed female). Different letters above the bars denote significant differences among treatments at P = 0.05.

In addition, warming and precipitation had a non-significant effect on adult fresh mass, whereas females had higher fresh mass than males, regardless of whether the galls were intact or removed (Fig. 3). Gall removal significantly increased adult fresh mass, but the increase was stronger in males than in females, as indicated by the significant interaction between gall and sex (Table 2).

## Discussion

Our study shows that warming significantly affected the mortality rate and the timing of adult emergence, but these warming effects depended on the amount of precipitation. Moreover, the results also indicate that the direction and magnitude of the warming effect on mortality rate and timing of emergence depended on whether the galls were intact or removed, and that the precipitation effect was significant on both the mortality and the timing when the galls were intact but non-significant when the galls were removed. These findings not only indicate that the effect of altered environmental conditions on species performance was significantly mediated by galls, but also suggest that the galling-making habit may improve *U*. *stylata*’s adaptability to fluctuating spring climate at the Tibetan Plateau.

The finding of warming increasing the survival and advancing the timing of emergence confirms our expectations since the physiological process rates of ectoderm insect activities are largely dependent upon external environmental temperature, which is often positively associated with enzyme activities^38^,^39^. The warming effect on species performance found here is consistent with results of many previous studies addressing insect survival and phenology^12^. However, the positive effect of warming occurs theoretically only when other factors that are responsible for physiological processes are not limited^11^. For example, warming should increase the energy demand and nutrient uptake of animals^33^, and hence, if nutrient level is low enough, this may slow down animal metabolic rates. In this study, the larval maggots almost reached their maximum body size at the end of the growing season when the plants die off, and larval maggots do not consume plant tissues in the next spring (X. Xi and S. Sun; personal observation). This suggests that the larval response to warming likely was not limited by nutrients. Other potential factors that can confound the warming effect include photoperiod and water availability^26^. Several studies showed an interaction effect between temperature and photoperiod on animal phenology^3^, in which insects were inactive despite a temperature increase in the season of hibernation. The hibernating maggots of our study species are covered by galls, which usually are 5 mm to 10 mm thick and may totally prevent light penetration.

We have shown that precipitation significantly interacted with warming to affect the performance of the studied species. Water is an essential element of all animal body components and metabolic activity, and precipitation is a major signal for many insect species living in dry areas^40^. For example, in tropical savanna and desert systems, many beetle species become active only after a large precipitation event, and then they complete growth, development, and reproduction in a short rainy season^41^. Importantly, the precipitation effect was stronger in warmed than in the ambient temperature treatment for the maggots with intact galls. This indicates that the larval maggots are more likely to survive and emerge earlier under warmer and more humid conditions. Such species responses to warming and precipitation could help to adapt to environmental change in the spring of the Tibetan Plateau. Previous climatic records show that precipitation in spring (April to June) fluctuates considerably with a maximum of 220 mm and minimum of 76 mm, and with less than 100 mm in eleven out of 53 years (data from Hongyuan County climate station). This indicates that spring drought is not unusual at the study site, which would be detrimental to both plant and animal activities. Indeed, modeling work shows that the spring greening of the plateau plants is not only driven by temperature but also triggered by precipitation^36^,^42^. Thus, such plastic responses to precipitation and temperature might help synchronize the timings of fly emergence and its host plant flowering.

The comparison of *U*. *stylata* performance in the gall-intact and gall-removed treatments shows that the galling-making habit plays a decisive role in the sensitivity of larval maggots to precipitation as well as in modulating the warming effect on the survival and timing of adult emergence. Specifically, the precipitation effects on both the mortality and the phenology disappeared when the galls were removed. Moreover, the warming effect on mortality rate varied in direction depending on whether galls were intact or removed. Galls mediated climate effects, and this particular function can be attributed to the properties of the gall tissue. The galls mainly consist of plant fibers, and after plants die off, they are usually well insulated against temperature fluctuations outside, unless they get wet^43^. Consequently, when the larvae are living within galls, they can detect the humidity of gall tissues; when they are naked they hardly ‘sense’ the precipitation intensity (presumably because of quick water loss and ‘transient feeling’), as reflected by our results. As noted above, having galls and sensing precipitation is beneficial to the species for survival and phenological adaptation, but it can also be costly^44^. For example, the gall formation needs the costs of the larval physiological processes to manipulate plant nutrient transport and cell division^32^. In extremely dry years, having galls may cause a higher mortality rate because dry galls could be too compact for adults to emerge. Consistently, our results show that galls significantly decreased the adult fresh mass and induced a high mortality rate in the reduced precipitation treatment. It should be noted that it is not clear why males and females differed in their responses in the timing of emergence and adult fresh mass to the treatment factors. The differences may have unknown consequences for the fitness of the focal species, which needs further exploration.

In summary, we revealed that warming and altered precipitation interactively affected the mortality rate and the timing of adult emergence in spring for *Urophora stylata* in an alpine meadow of the Tibetan Plateau. Moreover, our results also demonstrated the important role of gall-making habit in mediating the response of the studied species to climate change. These results collectively suggest that the effect of elevated temperature on the performance of species may depend on other environmental conditions, such as variations in precipitation, and species traits like the formation of galls. Long-term studies are needed to explore the interactive effects of multiple environmental change agents as well as that of species traits on species population dynamics in a changing world.

## Methods

### Study site

This study was conducted in Hongyuan Country (32°48′N, 102°33′E), Sichuan province, China, in the eastern part of the Tibetan Plateau. The altitude is ca. 3500 m above sea level, and the climate is characterized by a short and cool summer and a long and cold winter. Mean annual temperature is 0.9 °C, with the maximum monthly average temperature being 10.9 °C (July) and the minimum monthly average temperature being −10.3 °C (January); mean annual precipitation is 744 mm, which mainly occurs during May to August^45^. Meteorological data during 1961 and 2013 collected by the local climate station showed that the mean annual temperature has increased at a rate of 0.29 °C per decade during this time period^46^.

Alpine meadow is the major vegetation type in the study area. Plant coverage mostly exceeds 90% in the meadow. Sedges like *Kobresia setchwanensis* and *Blysmus sinocompressus* dominate lowland and high soil moisture communities, while forbs like *Potentilla anserina*, *Saussurea nigrescens*, and *Anemone trullifolia* var. *linearis* are dominant species in communities with relatively low soil moisture content.

Arthropod species, such as dung decomposers^47^, pollinators^48^, and herbivorous insects^49^ are diverse and abundant in the meadow.

### Focal species

*Urophora stylata* (Diptera: Tephritidae) is a common univoltine fly, which is a pre-dispersal seed predator species of *Carduus nutans* (Asteraceae). The plant species is a biannual forb occurring along the roadside and other disturbed sites in the study area. Height of mature plants is between 0.5 m and 1.5 m, and each plant individual bears 5 to 50 capitula, which flower from late May to late August.

Female flies oviposit in the capitula of host plants before flowering, and larval maggots feed on developing seeds within the capitula until pupating. The larvae usually form visible galls two weeks after oviposition, and then they continue to grow and develop within the galls. In late September before winter, the galls, together with the maggots, drop down to the ground surface, as a result of the die-off of aboveground plant parts. Larval maggots overwinter within the galls and then pupate and emerge out of the galls next spring. However, it is not unusual that the galls are found damaged and the maggots are exposed to external environments due to unknown reasons.

### The experiment

We conducted a three-factorial experiment involving three factors including artificial warming (ambient vs. warmed), manipulated precipitation (30% increase, average of the past 53 years, 30% decrease), and gall presence (gall intact vs. removed), resulting in 12 treatments in total.

We collected medium sized galls from the fenced meadow in late April 2015 when maggots living in capitula were inactive. There were 4.2 ± 0.6 (N = 20) maggots in each gall. For the treatments with intact galls, the galls were placed onto 48 plastic pots (Diameter = 29.8 cm, depth = 25 cm), in which soil collected from the same site was filled to depth of 27 cm. Each pot had 6 galls that were evenly distributed, and pots were replicated eight times, resulting in 288 galls in total (48 pots × 6 galls). For the treatments with galls removed, we carefully removed the gall tissues using a knife and collected 90 naked larval maggots, each of which was enclosed by a 2 × 2 cm paper bag to prevent too quick water loss from the larval bodies, as did by Charlet (1989) for larval weevils (*Cylindrocopturus adspersus*) that were moved out of flower stalks. Each of the six gall-removed treatments had 15 replicates (paper bags). All the paper bags of the same treatment were placed onto one pot, such as done for the gall-intact treatment.

Warming was achieved by placing the pots under a 1 m long 1600 W infrared heater that hang 1.2 m above the ground surface. This infrared heater raised the temperature by 1.9 °C on average during the experiment (Supporting materials, Fig. S1A). The average precipitation was calculated from daily precipitation between 1961 and 2013, as recorded by Hongyuan County climatic station. Precipitation was manipulated every third day by increasing (+30%) and decreasing (−30%) precipitation and comparing it with a control treatment with average precipitation of the past 53 years. For each time, the water (Supporting materials, Fig. S1B) was directly sprayed to the galls with at speed of in ca. 200 ml per min.

The experiment was started on 28-Apr-2015 in a fenced plot within a typical alpine meadow. On 31-May-2015 before adult emergence, we enclosed all the galls as well as the paper bags and associated naked maggots using bags made of steel screen with the mesh size of 2 × 2 mm. During the experiment, the paper bags were replaced for the naked maggots at the same frequency as for precipitation manipulation.

Starting on 01-Jun-2015, we checked every day if there was a newly emerged adult fly in the steel bags. Once found, the adult was put into a 2 ml plastic tube, which was kept at 0 °C in a refrigerator after being weighed. We recorded the emergence day as the days after the beginning of the experiment for each gall (larva) and each paper bag. Ten days after the last flies had emerged from the galls, we removed galls or bags and found that all the larvae or pupae were dead in the galls or bags.

### Data analysis

The normality of the data on each variable was tested before analysis. Nested Four-way ANOVAs (individual maggots nested in galls) were used to determine the effect of warming, precipitation, gall, and sex on the on the timing of emergence and fresh body mass of the adult flies. Sex was included as factor because males and female flies largely differ in life-history traits (see also ref. 50). *Post hoc* Tukey HSD tests were employed to determine difference between treatments whenever a significant effect (P < 0.05) was found. General linear model (GLM; with binomial errors) was employed to determine the effects of warming, precipitation, and gall on the mortality rates of the larval maggots. As larval male and females were visually indistinguishable, sex was not included as a treatment factor here. “glht” within “multcomp” package was used to conduct *post hoc* tests after GLM. All the data analyses were performed by R^51^.

## Additional Information

**How to cite this article**: Xi, X. *et al*. Experimental warming and precipitation interactively modulate the mortality rate and timing of spring emergence of a gallmaking Tephritid fly. *Sci. Rep.* **6**, 32284; doi: 10.1038/srep32284 (2016).

## Supplementary Material

Supplementary Information

## Figures and Tables

**Table 1:** Results of generalized linear model (with binomial errors) showing the effects of warming (ambient, warmed (ambient +1.9 °C)), precipitation (increased (30% increased), average (average of past 53 years), and decreased (30% decreased)), and the presence of galls (intact, removed) as well as their interactions on the mortality rate of larval maggots at the end of the experiment.

	d.f.	χ^2^	P
Gall	1	1844	**0**.**046**
Warming	1	1834	**0**.**001**
Precipitation	2	1583	**<0**.**001**
Gall × Warming	1	1583	0.670
Gall × Precipitation	2	1547	**<0**.**001**
Warming × Precipitation	2	1530	**<0**.**001**
Gall × Warming × Precipitation	2	1527	0.180
Residual	1333	1913	

**Table 2:** Results of ANOVA showing the effects of warming (W, ambient, warmed (ambient +1.9 °C)), Precipitation (P, increased (30% increased), average (average of past 53 years), and decreased (30% decreased)), the presence of galls (G, intact, removed), and sex (S) as well as their interactions on timing of adult fly emergence and adult fresh mass.

	d.f.	Timing of emergence			Adult fresh mass		
		SS	F	P	SS	F	P
G	1	2860	214	**<0**.**001**	14	63.6	**<0**.**001**
W	1	215	16	**<0**.**001**	0.3	1.3	0.25
P	2	2799	105	**<0**.**001**	0.3	0.8	0.47
S	1	4040	303	**<0**.**001**	163	745	**<0**.**001**
G × W	1	227	17	**<0**.**001**	0.2	0.8	0.36
G × P	2	404	15	**<0**.**001**	0.14	0.32	0.73
W × P	2	1334	50	**<0**.**001**	0.31	0.7	0.5
G × S	1	71	5	**0**.**022**	2.1	3.3	**0**.**002**
W × S	1	57	4	**0**.**030**	0.02	0.3	0.94
P × S	2	69	3	0.078	0.26	3.3	0.55
G × W × P	2	349	13	**<0**.**001**	0.19	0.08	0.65
G × W × S	1	29	2	0.140	0.01	0.005	0.93
G × P × S	2	6	0.06	0.810	0.87	0.06	0.14
W × P × S	2	92	5.90	**0**.**030**	0.63	1.5	0.2
G × W × P × S	2	69	2.57	0.080	0.28	0.6	0.5
Residuals	239	3186			52		
