# Supplementary material for: Experimental warming and precipitation interactively modulate the mortality rate and timing of spring emergence of a gallmaking Tephritid fly
Source: Sci Rep. 2016 Aug 31;6:32284. doi: 10.1038/srep32284 (PMC5006085; doi:10.1038/srep32284)

Experimental warming and precipitation interactively modulate the mortality rate and timing of spring emergence of a gallmaking Tephritid fly

Xinqiang Xi[1], Dongbo Li[1], Youhong Peng[2], Nico Eisenhauer[3,4], Shucun Sun[1,2]

1 Department of Ecology, School of Life Sciences, Nanjing University, 163 Xianlindadao Avenue, Nanjing 210023, China

2 ECORES Lab, Chengdu Institute of Biology, Chinese Academy of Sciences, Chengdu 610041, China

3 German Centre for Integrative Biodiversity Research (iDiv) Halle-Jena-Leipzig, Deutscher Platz 5e, 04103 Leipzig, Germany

4 Institute for Biology, Leipzig University, Johannisallee 21, 04103 Leipzig, Germany

Corresponding author: Shucun Sun

Email: shcs@nju.edu.cn

Supporting information

Figure S1 Variation in daily temperature (A) and water volume added in 30% increased precipitation, average (average of past 53 years), and 30% decreased precipitation treatments (B) during the experiment.

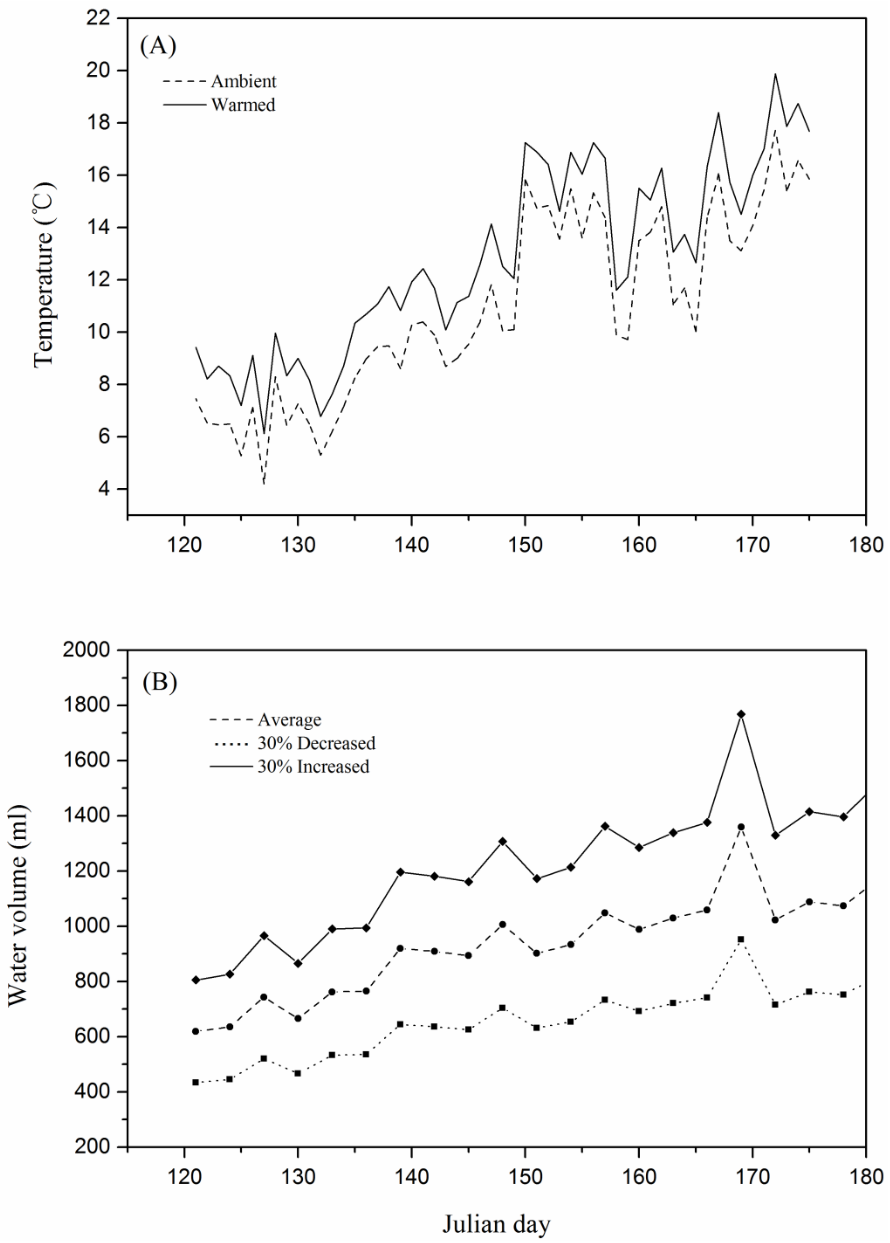

Supplement: Supplementary Information [file srep32284-s1.pdf]
